# Compensatory Base Changes in ITS2 Secondary Structures Correlate with the Biological Species Concept Despite Intragenomic Variability in ITS2 Sequences – A Proof of Concept

**DOI:** 10.1371/journal.pone.0066726

**Published:** 2013-06-24

**Authors:** Matthias Wolf, Shilin Chen, Jingyuan Song, Markus Ankenbrand, Tobias Müller

**Affiliations:** 1 Department of Bioinformatics, Biocenter, University of Würzburg, Würzburg, Germany; 2 Institute of Medicinal Plant Development, Chinese Academy of Medical Sciences, Peking Union Medical College, Beijing, China; University of California Riverside, United States of America

## Abstract

Compensatory base changes (CBCs) in internal transcribed spacer 2 (ITS2) rDNA secondary structures correlate with Ernst Mayr’s biological species concept. This hypothesis also referred to as the CBC species concept recently was subjected to large-scale testing, indicating two distinct probabilities. (1) If there is a CBC then there are two different species with a probability of ∼0.93. (2) If there is no CBC then there is the same species with a probability of ∼0.76. In ITS2 research, however, the main problem is the multicopy nature of ITS2 sequences. Most recently, 454 pyrosequencing data have been used to characterize more than 5000 intragenomic variations of ITS2 regions from 178 plant species, demonstrating that mutation of ITS2 is frequent, with a mean of 35 variants per species, respectively per individual organism. In this study, using those 454 data, the CBC criterion is reconsidered in the light of intragenomic variability, a proof of concept, a necessary criterion, expecting no intragenomic CBCs in variant ITS2 copies. In accordance with the CBC species concept, we could demonstrate that the probability that there is no intragenomic CBC is ∼0.99.

## Introduction

**Figure 1:**
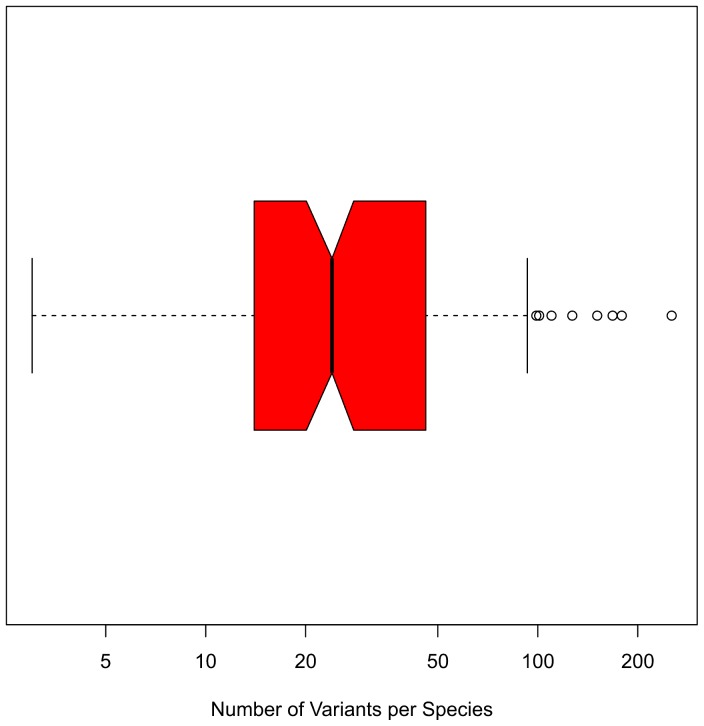
Boxplot of the number of intragenomic ITS2 variants per species on a log scale. The median number of variants is 23, while this number ranges from 1 up to 253 variants. Due to the right skewed distribution the mean number of variants is ∼35.

Compensatory base changes (CBCs) in internal transcribed spacer 2 (ITS2) secondary structures correlate with Ernst Mayr’s biological species concept from the 1940s. CBCs occur in a paired region of a primary RNA transcript when both nucleotides of a paired site mutate, while the pairing itself is maintained (e.g., G-C mutates to A-U) [1]. As cited verbatim already several times here again we introduce that according to Coleman and Vacquier [2], ‘‘… in all […] eukaryote groups where a broad array of species has been compared for both [rDNA] ITS2 sequence secondary structure and tested for any vestige of interspecies sexual compatibility, an interesting correlation has been found. When sufficient evolutionary distance has accumulated to produce even one CBC in the relatively conserved pairing positions of the ITS2 transcript secondary structure, taxa differing by the CBC are observed experimentally to be totally incapable of intercrossing’’ (see also [3], [4], [5], [6]). This hypothesis, also referred to as the CBC criterion or the CBC species concept was subjected to large-scale testing by Müller et al. [7], using the ITS2 database [8], [9], [10], [11], [12], which currently holds ∼300.000 ITS2 secondary structures, and the 4SALE program for synchronous sequence and secondary structure alignment and editing [13], [14]. The result of this comprehensive analysis indicated two distinct probabilities. (1) If there is a CBC then there are two different species with a probability of ∼93%. (2) If there is no CBC then there is the same species with a probability of ∼76% (both probabilities have been obtained on all four ITS2 helices, see below). Although the confidence in distinguishing species (if there is a CBC) is much higher than in merging them (if there is no CBC), both correlations (with more than 100 citations to [7]) have been used in practice. Counting for CBCs is possible despite high sequence variability because the ITS2 exhibits a common core of RNA secondary structure throughout the Eukaryota consisting of four helices, the third being the longest [15], [16], [17]. [This conserved ITS2 secondary structure is also one reason why in ITS2 sequence-structure phylogenetics including RNA secondary structures improves accuracy and robustness in reconstruction of phylogenetic trees [18]]. The ‘Faculty of 1000’ (in a comment by Richard Frankham, 2009) named CBCs between ITS2 sequence-structure pairs the ‘Holy Grail’ for molecular taxonomy [19]. However, “ITS2 is a double-edged tool for eukaryotic evolutionary comparisons” [6]. With all their benefits and pitfalls ITS2 sequences are used in phylogenetics, barcoding, metagenomics, and even DNA chip technologies (e.g. [20], [21]). In all these research fields the main problem is the multicopy nature of ITS2 sequences (and their concerted evolution) which currently fuels the controversy about the utility of ITS2 sequences (reviewed in [22]). Most recently, sequence-tagged [454] pyrosequencing and genome-wide analyses have been used to characterize intra-genomic variations of ITS2 regions from 178 plant species [23]. Song et al. (2012) “discovered that mutation of ITS2 is frequent, with a mean of 35 variants per species (Fig. 1). [However,] on average, three of the most abundant variants make up 91% of all ITS2 copies. […] DNA barcoding gap analysis showed that the intra-genomic distances were markedly smaller than those of the intra-specific or inter-specific variants. When each of [more than 5000] variants were examined for its species discrimination efficiency, a 97% success rate was obtained at the species level. Identification of identical ITS2 variants across intra-generic or inter-generic species revealed complex species evolutionary history, possibly, horizontal gene transfer and ancestral hybridization. Although intra-genomic multiple variants are frequently found within each genome, the usage of the major variants alone is sufficient for phylogeny [re]construction and species determination in most cases. Furthermore, the inclusion of minor variants further improves the resolution of species identification.” Today the utility of ITS2 sequences (and their secondary structures) in barcoding and phylogenetics is undoubted (e.g., [18], [20], [24], [25], [26], [27]); nevertheless the CBC criterion has not been evaluated in the light of intragenomic variability - a proof of concept, a necessary criterion - expecting no intragenomic CBCs in variant ITS2 copies.

## Materials and Methods

### Taxon Sampling

We analyzed 5543 intragenomic variants of the ITS2 from 247 samples from 178 species (i.e., some species are intermixed by several individuals) of 76 genera belonging to 44 families of angiosperms, gymnosperms, and ferns [23]. These samples included the 5 species (maize, Arabidopsis, poplar, and two rice species) with publicly available whole genome sequences, as well as the plant materials listed in Chinese Pharmacopoeia, which possesses medical importance. In addition, most genera have economical values, such as *Citrus*, *Panax*, *Dendrobium*, *Pinus,* etc. (cf. [23]).

### Sequence Analysis

ITS2 sequences were annotated according to Keller et al. [28]. All secondary structures were obtained from the ITS2 database [8], [9], [10], [11], [12]. Structures were predicted by either direct fold (energy minimization) or homology modelling [29]. Sequences and their individual secondary structures were synchronously aligned making use of an ITS2 sequence-structure specific scoring matrix [13]. All statistical analyses were calculated using the statistical framework R [30].

## Results

### Explorative Data Analysis

**Figure 2:**
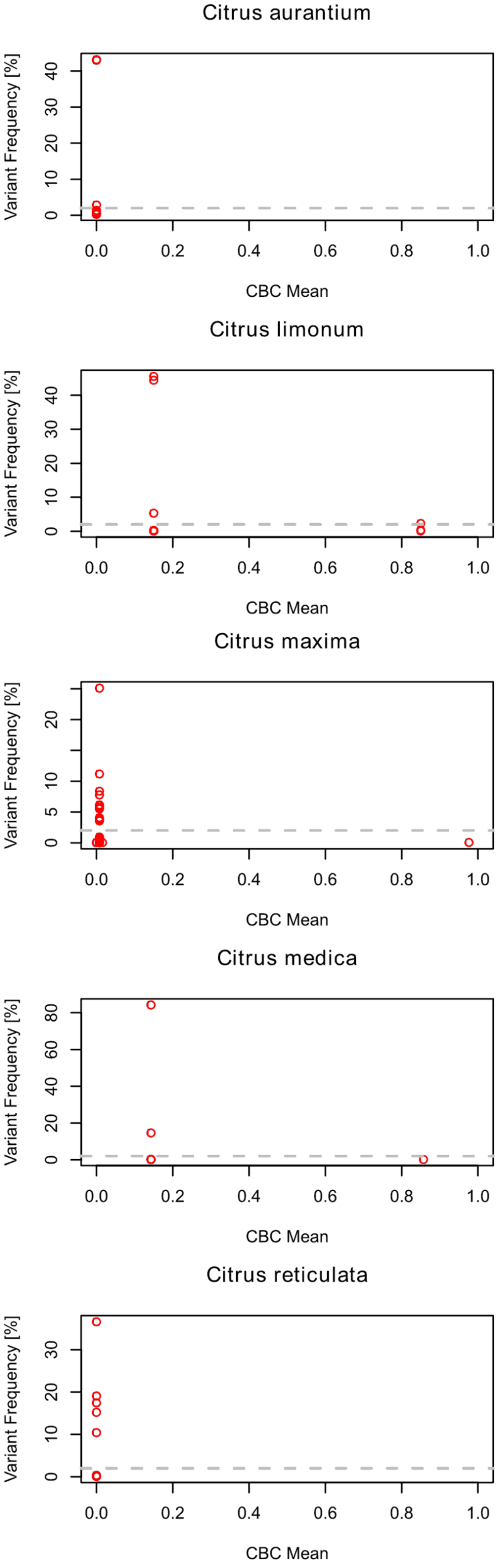
Scatterplot of all intragenomic ITS2 variants of five different species of the genus *Citrus*. The mean number of CBCs of one variant is plotted versus the variant frequencies. Typically, there are no CBCs between the variants as it is e.g. the case for *Citrus aurantium* and *Citrus reticulata*. Another typical behaviour is represented by all three other species, where one, several or a group of rare variants (<2%, as indicated by the dotted grey line) consistently show at least one CBC to the remaining ones. For all other 167 species investigated in this study see file S2.

Song et al. [23] provided more than 5000 intragenomic variants of the ITS2 from 247 individuals classified in 178 different species. All intragenomic variants of a species have been assigned with their occurrence frequencies. The variant with the highest frequency has been called main type. In this study, 167 main types secondary structures could be obtained by the ITS2 database either via homology modelling (102 species; average structure transfer ∼97%) or alternatively (65 species), by energy minimization [31]. Further, using the homology modelling approach according to Wolf et al. [29], in all 167 (out of 178) cases the main type secondary structure could be used as template for structure prediction of all other homologous intragenomic variants (average structure transfer ∼98%; file S1). All secondary structures of the considered variants show the typical core structure consisting of four helices with the third being the longest [17]. Now, based on the sequence-structure information 167 multiple sequence-structure alignments and CBC matrices have been calculated using the algorithms implemented in 4SALE [13], [14]. As exemplified for the genus *Citrus*, typically there are no CBCs between the variants. However, in rare cases one, several or a group of variants consistently show at least one CBC to the remaining ones (Fig. 2).

**Figure 3:**
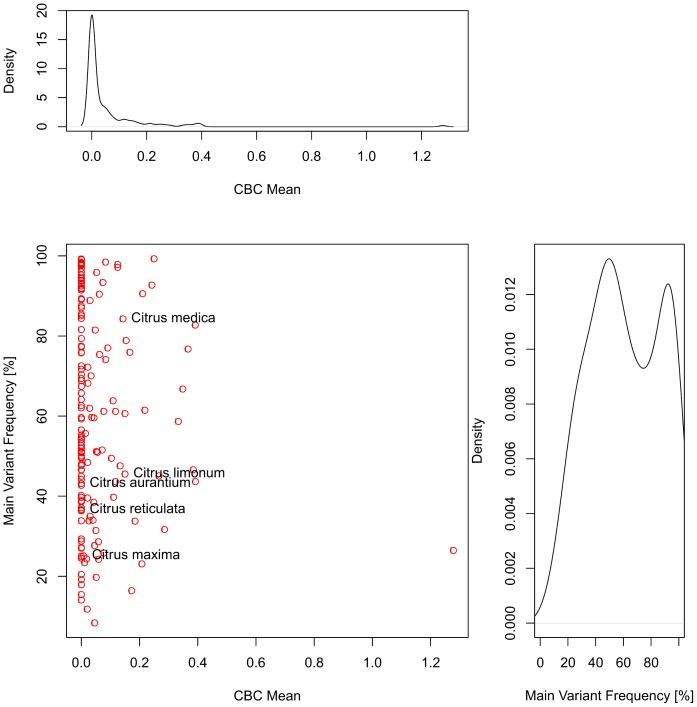
Scatterplot of the main intragenomic ITS2 variant of all species each. The frequency of the main variant is plotted versus the mean number of CBCs as compared to all remaining variants of the species. All 167 species are investigated. According to figure 2, exemplarily different species of the genus *Citrus* are highlighted right to the respective data point. For example the main variant of *Citrus medica* occurs with a frequency of ∼84%. Three remaining variants (cf. Fig. 2) together occur in 16% and show 0.2 CBCs on average as compared to the main type. Most data points are close to zero and show no CBCs between different variants. Additionally, densities of the main variant frequencies and CBC means are given above and right to the respective margins. Mainly, there are one or two main variants per species.

The main type of course is of the highest biological interest, because this variant might typically be used in real life work. Therefore we focused on the number of CBCs between the main type and the remaining variants, where generally no CBCs could be detected (Fig. 3).

### The CBC Species Concept – a Necessary Criterion

In fact there are intragenomic CBCs – however, there are not many. First of all, (1) we followed the algorithm of Müller et al [7] and picked randomly a tuple of different intragenomic variants and counted the occurrence of one or more CBCs. This is done for all available species. Finally, the whole procedure is repeated 1000 times and the results are averaged. This procedure yielded 0.0669 intragenomic CBCs. As a second method (2) we directly averaged the whole variant CBC matrix (presence/absence) for each species. The average of these averages is 0.0666 intragenomic CBCs and demonstrates that method (1) as expected converges to method (2). However, both methods do not consider the variant frequencies. Therefore, we finally extend the second method (3) taking into account the variant frequencies as provided by Song et al. [23]. Integrating these frequencies into a weighted averaging procedure yielded 0.0001 intragenomic CBCs. In our opinion, this last method best mimics the CBC frequency as expected in everyday work when using the CBC criterion and is now further used throughout the manuscript. All CBC counts are summarized in Table 1.

**Table 1:** Intragenomic CBC distribution.

Count Method	CBC = 0	CBC >0
Method 1 (sampling)	0.9331	0.0669
Method 2 (averaging)	0.9334	0.0666
Method 3 (weighted average)	0.9999	0.0001

According to the law of large numbers [32] the relative frequencies converge to the probability and the averages converge to the expectation. Here, the calculated average 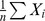 converges to the expectation of X, where X denotes the Bernoulli random variable, which is 1 if we observe one or more CBCs and 0 else. Now, the expectation of this indicator variable X equals to the probability of a CBC. Because we only sample our data in one species, the average converges to the probability of a CBC in one species.

Thus we have:
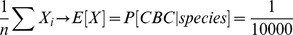



In other words, the probability that there is no intragenomic CBC is 0.9999.

## Discussion and Conclusions

Compensatory base changes (CBCs) in internal transcribed spacer 2 (ITS2) secondary structures correlate with the biological species concept [7]. If there is a CBC then there are two different species with a probability of ∼0.93. If there is no CBC then there is the same species with a probability of ∼0.76. There is no causal relationship between a CBC and speciation. There is just a correlation. The correlation works well in practice, at least in one direction; however, the multicopy nature of ITS2 was not part of the concept; concerted evolution was assumed and intragenomic data have not been available. Clearly, in accordance with the CBC criterion there must be no intragenomic CBC – a proof of concept and a necessary criterion. Therefore, in this study, with appropriately associated data available for the first time, for 178 plant species the CBC criterion was reconsidered in the light of intragenomic variability. Mutation of ITS2 is frequent, with a mean of 35 variants per species. However, on average, three of the most abundant variants make up>90% of all intragenomic ITS2 copies. There are intragenomic CBCs, however there are not many. In fact we demonstrated that the probability that there is no intragenomic CBC is ∼0.99 (99.99%). Speciation is a continuous process and species incessantly evolve, which obviously causes an incomplete concerted evolution. Rarely, an incomplete concerted evolution could be explained by molecular fossils. Of course the processes of concerted evolution as well as the complex evolutionary history of molecular fossils need to be investigated further. Is a rare intragenomic ITS2 variant in one species the main variant in a closely related species? Does a rare intragenomic CBC distinguish an “intragenomic species”? What are the differences between intragenomic variants of one individual organism in contrast to intragenomic variants of a species (for different numbers of ITS2 variants per organism see file S3)? Ploidy also could affect the number of observed CBCs. However, this potential bias is already included in the 0.99 probability that there is no intragenomic CBC. Moreover, intragenomic variation is not necessary higher the higher the number of chromosomes [23]. Last but not least, still the question remains, what really is a species? Most species concepts, e.g. the CBC species concept, are just indicator hypotheses, not definitions. The data available may already provide some insights to those questions (cf. [23]), but those questions are beyond the scope of this study. Nevertheless, if there is a CBC, the CBC species concept could be used in distinguishing species according to Müller et al. [7]. In other words, CBCs in ITS2 secondary structures correlate with the biological species concept despite intragenomic variability in ITS2 sequences.

## Supporting Information

File S1 **Secondary structure prediction.** The table provides the success rate in structure prediction via direct fold or homology modelling concerning 178 species. Sixty five main type secondary structures were obtained by direct fold (energy minimization). One hundred and two main type secondary structures were obtained by homology modelling. Template secondary structures and their gi numbers were obtained from the ITS2 database. Species names have been identical for template/target pairs in 75 cases (indicated by an asterisk, 4 synonyms). In 27 cases templates were obtained from closely related species classified in the same genus (3 synonyms). The one hundred and sixty seven main type secondary structures were used for homology modelling of intragenomic variants. In summary, 167 (out of 178) species for which high quality secondary structures could be predicted have been used for the intragenomic CBC analysis. For eleven species no secondary structures could be obtained (i.e., secondary structures deviate from the common core structure; further studies are needed). In two species for one (‘&’) or two (‘$’) intragenomic variants no secondary structure could be obtained.
(DOC)

File S2 **Scatterplots of all intragenomic ITS2 variants of all 167 different species investigated in this study.** The mean number of CBCs of one variant is plotted versus the variant frequencies.
(TIF)

File S3 **Boxplot of different numbers of variants per organism, each classified in the same species.**
(TIF)
