# Supplementary material for: Compensatory Base Changes in ITS2 Secondary Structures Correlate with the Biological Species Concept Despite Intragenomic Variability in ITS2 Sequences – A Proof of Concept
Source: PLoS One. 2013 Jun 24;8(6):e66726. doi: 10.1371/journal.pone.0066726 (PMC3691174; doi:10.1371/journal.pone.0066726)
Supplement: File S1 — Secondary structure prediction. The table provides the success rate in structure prediction via direct fold or homology modelling concerning 178 species. Sixty five main type secondary structures were obtained by direct fold (energy minimization). One hundred and two main type secondary structures were obtained by homology modelling. Template secondary structures and their gi numbers were obtained from the ITS2 database. Species names have been identical for template/target pairs in 75 cases (indicated by an asterisk, 4 synonyms). In 27 cases templates were obtained from closely related species classified in the same genus (3 synonyms). The one hundred and sixty seven main type secondary structures were used for homology modelling of intragenomic variants. In summary, 167 (out of 178) species for which high quality secondary structures could be predicted have been used for the intragenomic CBC analysis. For eleven species no secondary structures could be obtained (i.e., secondary structures deviate from the common core structure; further studies are needed). In two species for one (‘&’) or two (‘$’) intragenomic variants no secondary structure could be obtained. (DOC) [file pone.0066726.s001.doc]

| Species | Folding | Template |
| --- | --- | --- |
| *Acorus calamus* | direct |  |
| *Acorus gramineus* | direct |  |
| *Allium tuberosum* | direct |  |
| *Aloe vera* | homology | 7230664* |
| *Alpinia galanga* | direct |  |
| *Alpinia hainanensis* | homology | 311700567* |
| *Alpinia zerumbet* | homology | 19310146* |
| *Amygdalus persica* | direct |  |
| *Amygdalus triloba* | direct |  |
| *Angelica dahurica* | direct |  |
| *Angelica decursiva* | homology | 82590166* |
| *Angelica sinensis* | homology | 283771435* |
| *Arabidopsis thaliana* | direct |  |
| *Ardisia crenata* | direct |  |
| *Ardisia japonica* | direct |  |
| *Armeniaca sibirica* | homology | 13430142, *Prunus armeniaca* |
| *Armeniaca vulgaris* | homology | 8100526, *Prunus mandshurica* |
| *Artemisia annua* | homology | 220901174* |
| *Artemisia argyi* | homology | 220901175* |
| *Artemisia capillaris* | homology | 313770890, *Artemisia scoparia* |
| *Artemisia gmelinii* | homology | 238695927* |
| *Artemisia lavandulaefolia* | homology | 238695963* |
| *Asparagus cochinchinensis* | homology | 299767232* |
| *Asparagus schoberioides* | homology | 299767233, *Asparagus filicinus* |
| *Asparagus trichophyllus* | homology | 289467498, *Asparagus officinalis* |
| *Aster ageratoides* | homology | 320001167* |
| *Aster tataricus* | homology | 313770893* |
| *Astragalus chinensis* | direct |  |
| *Astragalus hancockii* | homology | 300089201, *Astragalus trichopodus* |
| *Astragalus mongholicus* | direct |  |
| *Boehmeria nivea* | direct |  |
| *Brucea javanica* | direct |  |
| *Brucea mollis* | direct |  |
| *Celosia argentea* | direct |  |
| *Celosia cristata* | direct |  |
| *Cerasus glandulosa* | homology | 13430113* |
| *Cerasus japonica* | homology | 5163374* |
| *Cerasus tomentosa* | homology | 12002909, *Prunus tomentosa* |
| *Cimicifuga dahurica* | direct |  |
| *Cimicifuga foetida* | direct |  |
| *Cirsium japonicum* | homology | 313770865* |
| *Cirsium setosum* | direct |  |
| *Citrus aurantium* | homology | 224471374* |
| *Citrus limonum* | direct |  |
| *Citrus maxima* | direct |  |
| *Citrus medica* | direct |  |
| *Citrus reticulata* | direct |  |
| *Clerodendrum bungei* | homology | 238695979* |
| *Clerodendrum cytophyllum* | homology | 33320122, *Clerodendrum trichotomum* |
| *Clerodendrum japonicum* | homology | 238695978* |
| *Cryptotaenia japonica* | homology | 46486760* |
| *Cynanchum atratum* | homology | 238695974, *Vincetoxicum atratum* |
| *Cynanchum paniculatum* | homology | 238695974, *Vincetoxicum atratum* |
| *Cynanchum stauntonii* | homology | 222139377* |
| *Datura arborea* | not available |  |
| *Datura metel* | direct |  |
| *Daucus carota* | homology | 18366* |
| *Dendrobium capillipes* | not available |  |
| *Dendrobium catenatum* | homology | 14993605, *Dendrobium officinale* |
| *Dendrobium chrysanthum* | homology | 300244433* |
| *Dendrobium chrysotoxum* | homology | 14993593* |
| *Dendrobium crepidatum* | direct |  |
| *Dendrobium crystallinum* | homology | 300250566, *Dendrobium bensoniae* |
| *Dendrobium cucullatum* | homology | 300244441, *Dendrobium aphyllum* |
| *Dendrobium denneanum* | direct |  |
| *Dendrobium devonianum* | not available |  |
| *Dendrobium gratiosissimum* | homology | 14993591, *Dendrobium aduncum* |
| *Dendrobium loddigesii* | homology | 14993600* |
| *Dendrobium nobile* | homology | 300244439* |
| *Dendrobium pendulum* | homology | 70795241* |
| *Dendrobium polyanthum* | homology | 300250743, *Dendrobium primulinum* |
| *Dendrobium spatella* | homology | 33088263, *Dendrobium confusum* |
| *Dendrobium trigonopus* | homology | 70795244* |
| *Dendrobium wardianum* | homology | 70795239* |
| *Dendrobium williamsonii* | homology | 14993606* |
| *Dichroa febrifuga* | homology | 238695977* |
| *Eleutherococcus giraldii* | homology | 46486711* |
| *Eleutherococcus nodiflorus* | homology | 2826482* |
| *Eleutherococcus senticosus* | homology | 300068895* |
| *Ephedra equisetina* | not available |  |
| *Ephedra sinica* | not available |  |
| *Epimedium acuminatum* | homology | 38349158, *Epimedium wushanense* |
| *Epimedium pubescens* | homology | 38349153* |
| *Epimedium sagittatum* | homology | 38349164* |
| *Eupatorium fortunei* | direct |  |
| *Euphorbia esula* | direct |  |
| *Euphorbia hirta* | not available |  |
| *Euphorbia pekinensis* | direct |  |
| *Flemingia lineata* | direct |  |
| *Flemingia macrophylla* | direct |  |
| *Foeniculum vulgare* | homology | 311294685* |
| *Gentiana macrophylla* | homology | 238695973, *Gentiana straminea* |
| *Gentiana manshurica* | homology | 261865079, *Gentiana scabra* |
| *Gentiana rigescens* | homology | 261865085* |
| *Gentiana straminea* | homology | 238695973* |
| *Ilex asprella* | direct |  |
| *Ilex cornuta* | direct |  |
| *Ilex pubilimba* | homology | 22796766, *Ilex goshiensis* |
| *Ilex rotunda* | homology | 24430011* |
| *Inula britanica* | homology | 313770879* |
| *Inula cappa* | homology | 148578042, *Duhaldea cappa* |
| *Inula helenium* | homology | 161105467* |
| *Ipomoea nil* | homology | 4588596* |
| *Ipomoea purpurea* | homology | 163963332* |
| *Ligusticum jeholense* | homology | 46486745* |
| *Ligusticum sinense cv. Chuanxiong* | homology | 46486758, *Ligusticum sinense* |
| *Ligusticum sinense* | homology | 46486758* |
| *Lilium pumilum* | homology | 5081336* |
| *Lilium tigrinum* | homology | 48926836, *Lilium sachalinense* |
| *Liriope spicata* | not available |  |
| *Lonicera confusa* | homology | 237687562* |
| *Lonicera japonica* | homology | 229621271, *Lonicera macranthoides* |
| *Lygodium japonicum* | not available |  |
| *Melicope pteleifolia* | direct |  |
| *Oryza sativa ssp. indica* | homology | 237784496* |
| *Oryza sativa ssp. japonica* | homology | 58652042* |
| *Paeonia anomala ssp. veitchii* | direct |  |
| *Paeonia lactiflora* | direct |  |
| *Paeonia ostii* | direct |  |
| *Panax ginseng* | homology | 89160943* |
| *Panax japonicus* | homology | 306754699* |
| *Panaxnotoginseng* | homology | 306754706* |
| *Panax quinquefolius* | homology | 306754710* |
| *Periploca sepium* | homology | 118640293, *Periploca graeca* |
| *Pinus bungeana* | direct |  |
| *Pinus ponderosa* | direct |  |
| *Pinus strobus* | direct |  |
| *Pinus wallichiana* | direct |  |
| *Piper longum* | homology | 238695910* |
| *Piper nigrum* | homology | 115393926* |
| *Polygonum chinense* | homology | 219944474* |
| *Populus trichocarpa*^&^ | homology | 5912284* |
| *Potentilla chinensis* | direct |  |
| *Potentilla fruticosa* | homology | 9392379, *Dasiphora fruticosa* |
| *Potentilla nivea* | direct |  |
| *Potentilla supina* | direct |  |
| *Prenanthes tatarinowii* | direct |  |
| *Pueraria montana var. lobata* | homology | 94449068* |
| *Pueraria peduncularis* | not available |  |
| *Rhus chinensis* | homology | 55977580* |
| *Rosa bella* | direct |  |
| *Rosa laevigata* | direct |  |
| *Rubus chingii* | direct |  |
| *Rubus parvifolius* | direct |  |
| *Santalum album* | homology | 299810760* |
| *Sedum emarginatum* | homology | 185813907* |
| *Sedum lineare* | homology | 26522918, *Sedum mexicanum* |
| *Sedum sarmentosum* | homology | 26522924, *Sedum lineare* |
| *Selaginella doederleinii* | direct |  |
| *Selaginella moellendorffii* | not available |  |
| *Selaginella uncinata* | direct |  |
| *Senna alata* | homology | 238696022* |
| *Senna obtusifolia* | direct |  |
| *Siegesbeckia glabrescens* | direct |  |
| *Siegesbeckia orientalis* | direct |  |
| *Siraitia grosvenorii* | homology | 259221480* |
| *Solanum lyratum*^$^ | homology | 7533161, *Solanum wallacei* |
| *Solanum nigrum* | not available |  |
| *Sophora flavescens* | direct |  |
| *Sophora japonica* | direct |  |
| *Sophora tonkinensis* | direct |  |
| *Stellaria vestita* | homology | 121300181* |
| *Stemmacantha uniflora* | direct |  |
| *Tetradium ruticarpum* | direct |  |
| *Torreya californica* | homology | 20339346* |
| *Torreya fargesii var. yunnanensis* | homology | 20339352, *Torreya nucifera* |
| *Torreya grandis* | direct |  |
| *Torreya nucifera* | homology | 20339352* |
| *Uncaria macrophylla* | homology | 302566823, *Uncaria sessilifructus* |
| *Uncaria sessilifructus* | homology | 302566823* |
| *Uncaria sinensis* | homology | 238695996* |
| *Verbena officinalis* | homology | 260618082* |
| *Veronicastrum axillare* | homology | 238696042* |
| *Veronicastrum stenostachyum* | homology | 14994015* |
| *Viola diffusa* | direct |  |
| *Viola philippica* | direct |  |
| *Zanthoxylum bungeanum* | direct |  |
| *Zea mays* | direct |  |
